# The role of long noncoding RNA myocardial infarction-associated transcript in hepatocellular carcinoma: Targeting miR-361-3p to regulate cell proliferation, invasion, and apoptosis

**DOI:** 10.1097/MD.0000000000047863

**Published:** 2026-03-27

**Authors:** Jie Tang, Hao Luo, Dongyun Hang, Qingyu Wang, Di Jin, Ming Xu, Liya Xu

**Affiliations:** a Department of Gastroenterology, Shanghai Pudong New Area People’s Hospital, Shanghai, China; b Department of Gastroenterology, Shanghai Hongkou District Jiangwan Hospital, Shanghai, China.

**Keywords:** cell apoptosis, cell invasion, cell proliferation, hepatocellular carcinoma, long noncoding RNA myocardial infarction-associated transcript, miR-361-3P

## Abstract

This study aims to investigate the effects of long noncoding RNA myocardial infarction-associated transcript (lncRNA MIAT) targeting miR-361-3p on the proliferation, invasion, and apoptosis of hepatocellular carcinoma (HCC) cells. The expression levels of lncRNA MIAT and miR-361-3p were examined in both cancerous and adjacent non-tumorous tissues of HCC patients. Additionally, their expression was analyzed in THLE-2 and MHCC97L cells. MHCC97L cells were transfected to observe changes in relevant mRNA expression and alterations in cell proliferation, invasion, and apoptosis. Compared with adjacent non-tumorous tissues, cancerous tissues exhibited increased levels of lncRNA MIAT mRNA and decreased levels of miR-361-3p (*P* < .05). Elevated transcription levels of lncRNA MIAT were more prevalent in patients with lymph node metastasis and advanced TNM staging (*P* < .05). In MHCC97L cells, compared with THLE-2 cells, there was an increase in lncRNA MIAT mRNA and a decrease in miR-361-3p (*P* < .05). LncRNA MIAT and miR-361-3p were found to have potential binding sites and exhibit target regulation. Cells in the miR-NC group showed no significant changes in all indicators compared with the negative control group (*P* > .05). Conversely, cells with low expression of lncRNA MIAT and cyclin D1 mRNA and decreased cell proliferation and invasion numbers showed increased expression of miR-361-3p and phosphatase and tensin homolog mRNA and higher apoptosis rates (*P* < .05). The group with elevated expression levels demonstrated contrasting patterns (*P* < .05). LncRNA MIAT is upregulated in HCC tissues. Silencing lncRNA MIAT can elevate miR-361-3p levels, inhibiting the proliferation and invasion of MHCC97L cell structures and promoting apoptosis.

## 1. Introduction

Globally, liver cancer claims approximately 350,000 lives annually, with nearly half of these cases originating in China, posing a severe threat to public health.^[1]^ Hepatocellular carcinoma (HCC) ranks as the most commonly diagnosed form of primary liver cancer. This condition is marked by low surgical resection rates and high postoperative recurrence and metastasis rates, significantly impacting the survival rates of patients with HCC.^[2]^ Furthermore, the underlying molecular mechanisms of HCC progression and invasion remain elusive. Therefore, exploring mechanisms that inhibit cell proliferation and promote apoptosis in HCC cells could present novel potential targets in HCC therapy. Recent studies have identified long noncoding RNAs (lncRNAs) as pivotal regulators in apoptosis and tumor cell invasion, akin to oncogenes or tumor suppressor genes.^[3]^ While numerous studies have assessed the impact of lncRNAs on the development and progression of HCC, detailed mechanisms of only a few lncRNAs have been thoroughly investigated. Research on the systematic identification and immunological characteristics of lncRNAs closely associated with HCC is still in its infancy.^[4]^

Consequently, understanding the impact of lncRNAs on the immune microenvironment of HCC and their molecular mechanisms is of utmost importance. Myocardial infarction-associated transcript (MIAT), a significant lncRNA related to cancer processes, exhibits abnormal expression in various tumors, including gastric, cervical, ovarian, and colorectal cancers.^[5–8]^ However, reports on the role of lncRNA MIAT in HCC are scarce. miR-361-3p, closely associated with the development and progression of HCC, has been found to impede the proliferation and invasion of HCC cells and encourage apoptosis when upregulated.^[9]^ Bioinformatics analysis suggests that lncRNA MIAT could potentially be a specific target gene of miR-361-3p. Hence, this study investigates lncRNA MIAT’s expression within HCC and examines its role in regulating miR-361-3p, affecting cell proliferation, invasion, and apoptosis in HCC cells, thereby offering new potential therapeutic targets for HCC treatment.

## 2. Materials and methods

### 2.1. Research cohort

Retrospective data analysis was performed for 45 individuals with HCC who received surgical treatment at our institution from January 2020 to December 2022. The average age was 61.31 ± 17.91 years. All patients met the diagnostic criteria outlined in the “Standards for the Diagnosis and Treatment of Primary Liver Cancer” (2017 edition), and this research adhered to the guidelines of the principles of the Declaration of Helsinki.^[10]^

### 2.2. Key reagents and equipment

The study utilized normal liver cells (THLE-2) and HCC cells (MHCC97L) sourced from the Kunming Cell Bank. Culture media comprised minimum essential medium and fetal bovine serum (Hyclone). Transfection reagents included a kit, negative control (siR-NC), lncRNA MIAT RNAi, lncRNA MIAT plasmid, miR-361-3p inhibitor, miR-361-3p mimic, and a dual-luciferase reporter assay kit (Shanghai Jikai Gene Chemical Technology Co., Ltd.). Primers for lncRNA MIAT (forward: 5′-TCCTTGCGAGACTTAAATGCGT-3′, reverse: 5′-GGACAAGCTCTGTAGCTGT-3′), miR-361-3p (forward: 5′-GAGAGATCTTGTGGGGAG-3′, reverse: 5′-CTGTGGAGTATGGAGGA-3′), phosphatase and tensin homolog (PTEN) (forward: 5′-TGCGTTGCACCTTATCCTT-3′, reverse: 5′-CTTCCTTCACAGTTTCG-3′), cyclin D1 (forward: 5′-CGTTCAAGGGGGATTAG-3′, reverse: 5′-CCCAGCACGGTACTTGC-3′), and β-actin (forward: 5′-TGGCACCCAGCACAATGAA-3′, reverse: 5′-CTAAGTCATAGTCCGCCTCA-3′) were used. Baosun Biotechnology Engineering Co., Ltd. (Dalian) produced fluorescent quantitative PCR reagents and RNA reverse transcription kits. Other materials included Trizol (Invitrogen), cell counting kit-8 (CCK-8, Amresco), Matrigel and 24-well Transwell chambers (Millipore), Annexin V/PI apoptosis detection kit (BD Biosciences), CO_2_ incubator (Thermo Revco), ELISA reader and flow cytometer (Beckman Coulter), and a real-time PCR system (Life Technologies).

### 2.3. Luciferase reporter assay

MHCC97L cells were transfected with lncRNA MIAT RNAi, lncRNA MIAT plasmid, miR-NC, miR-361-3p inhibitor, and miR-361-3p mimic. Luciferase activity was analyzed 48 hours post-transfection.

### 2.4. Cell culture and transfection

THLE-2 and MHCC97L cells were cultured under standard conditions for experimentation. MHCC97L cells were categorized into 4 groups: the negative control group, the miR-NC group, the lncRNA MIAT RNAi group (labeled as the low expression group), and the lncRNA MIAT plasmid group (labeled as the high expression group). Cells in the negative control group were untreated, while those in the miR-NC, low expression, and high expression groups were transfected with miR-NC, MIAT RNAi, and lncRNA MIAT plasmid, respectively, using the Lipofectamine method. Subsequent analyses were conducted 48 hours post-transfection.

### 2.5. RT-PCR analysis of mRNA expression

Cells treated according to Section 2.4 were seeded at 5 × 10^4^ cells per well in 6-well plates (3 mL per well). After 48 hours, RNA was extracted using Trizol, reverse transcribed into complementary deoxyribonucleic acid, and amplified in a 20 μL reaction mixture using a real-time PCR system. Relative levels of mRNA expression were determined by applying the 2^−ΔΔC*t*^ technique. Expression levels of lncRNA MIAT and miR-361-3p mRNA in cancerous tissues, adjacent non-tumorous tissues, THLE-2, and MHCC97L cells were also measured.

### 2.6. CCK-8 assay for cell proliferation

Following the method outlined in Section 2.4, cells were cultured in 96-well plates. After 44 hours, each well was supplemented with 10 μL of CCK-8 solution, and then it underwent an additional incubation period of 4 hours. Dimethyl sulfoxide (200 μL per well) was added, and the optical density (OD) values were measured using an ELISA reader. Cell proliferation rates were determined by comparing the OD value of the experimental group with that of the negative control group ([experimental group OD]/[negative control group OD]).

### 2.7. Transwell assay for cell migration

Cells treated as per Section 2.4 were seeded in 24-well Transwell chambers. After 48 hours, the chambers were fixed and treated with crystal violet. The stained cells were then rinsed and observed for enumeration through a microscope.

### 2.8. Flow cytometry for apoptosis detection

Cells processed according to Section 2.4 were seeded in 6-well plates. After 48 hours, they were resuspended in annexin V binding solution (1X) for setting the cell density at 1 × 10^6^/mL and analyzed for apoptosis using a flow cytometer.

### 2.9. Statistical analysis

Data were analyzed using SPSS software version 19.0. A *P*-value < .05 was deemed to indicate statistical significance.

## 3. Results

### 3.1. Expression of lncRNA MIAT and miR-361-3p mRNA in tissues

Relative to adjacent non-tumorous tissues, cancerous tissues exhibited increased levels of lncRNA MIAT mRNA and reduced levels of miR-361-3p (*P* < .05), as shown in Table 1.

**Table 1:** Expression of lncRNA MIAT and miR-361-3p mRNA in tissues.

Tissue type	lncRNA MIAT	miR-361-3p
Adjacent non-tumorous tissues	1.00 ± 0.18	1.00 ± 0.11
Cancerous tissues	1.96 ± 0.12*	0.57 ± 0.16*

lncRNA = long noncoding RNA, MIAT = myocardial infarction-associated transcript, miR = microRNA.

* Compared with THLE-2 cells, *P* < .05.

### 3.2. Correlation between lncRNA MIAT and clinical pathological characteristics

Patients were categorized into cohorts of high expression (25 cases) and low expression (20 cases), determined by the median expression level. The probability of high lncRNA MIAT expression was greater in patients with lymph node metastasis and advanced TNM staging (*P* < .05), as depicted in Table 2.

**Table 2:** LncRNA MIAT expression in HCC patients with different clinical pathological characteristics.

Factor	n	Low expression (%)	High expression (%)
Age			
≤60 yr	27	12 (44.44)	15 (55.56)
>60 yr	18	8 (44.44)	10 (55.56)
Gender			
Male	24	11 (45.83)	13 (54.17)
Female	21	9 (42.86)	12 (57.14)
Tumor diameter			
≤3 cm	26	10 (38.46)	16 (61.54)
>3 cm	19	10 (52.63)	9 (47.37)
Metastasis*			
Lymph node	28	8 (28.57)	20 (71.43)
No lymph node	17	12 (70.59)	5 (29.41)
TNM stage*			
I/II	19	13 (68.42)	6 (31.58)
III	26	7 (26.92)	19 (73.08)

HCC = hepatocellular carcinoma, lncRNA = long noncoding RNA, MIAT = myocardial infarction-associated transcript.

* Compared with high expression, *P* < .05.

### 3.3. Expression of lncRNA MIAT and miR-361-3p mRNA in cells

Compared with THLE-2 cells, MHCC97L cells showed increased lncRNA MIAT mRNA levels and decreased miR-361-3p levels (*P* < .05), as detailed in Table 3.

**Table 3:** Expression of lncRNA MIAT and miR-361-3p mRNA in cells.

Cell type	lncRNA MIAT	miR-361-3p
THLE-2 cells	1.00 ± 0.24	1.00 ± 0.13
MHCC97L cells	1.84 ± 0.17*	0.68 ± 0.10*

lncRNA = long noncoding RNA, MIAT = myocardial infarction-associated transcript, miR = microRNA.

* Compared with adjacent non-tumorous tissues, *P* < .05.

### 3.4. Target regulation of miR-361-3p by lncRNA MIAT

**Figure 1.**
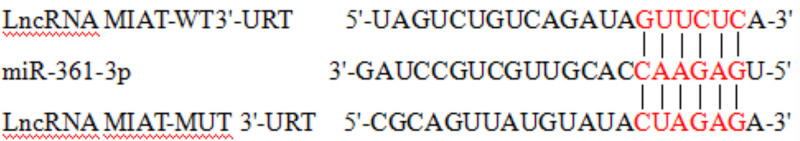
Target regulation of miR-361-3p by lncRNA MIAT. lncRNA = long noncoding RNA, MIAT = myocardial infarction-associated transcript, miR = microRNA, MUT = mutant.

LncRNA MIAT was found to have possible binding sites with miR-361-3p; transfection with miR-361-3p mimic notably diminished lncRNA MIAT-WT’s functionality, without impacting lncRNA MIAT-mutant, while transfection with miR-361-3p inhibitor had the opposite effect. This is illustrated in Figure 1 and Table 4.

**Table 4:** Dual-luciferase reporter assay results.

Group	lncRNA MIAT-WT	lncRNA MIAT-MUT
Control group	1.00 ± 0.15	1.00 ± 0.11
miR-NC group	1.04 ± 0.13	1.09 ± 0.12
miR-361-3p inhibitor group	1.00 ± 0.19	0.68 ± 0.04*,†
miR-361-3p mimic group	1.96 ± 0.20*^,^†^,^‡	0.98 ± 0.14

lncRNA = long noncoding RNA, MIAT = myocardial infarction-associated transcript, miR = microRNA, MUT = mutant.

* Compared with the control group, *P* < .05.

† Compared with the miR-NC group, *P* < .05.

‡ Compared with the miR-361-3p inhibitor group, *P* < .05.

### 3.5. Comparison of lncRNA MIAT, miR-361-3p, PTEN, and cyclin D1 mRNA expression in different cell groups

No notable disparity in indicators was observed when comparing the negative control with the miR-NC groups (*P* > .05). Compared with the negative control group, the low expression group showed decreased expression of lncRNA MIAT and cyclin D1 mRNA and increased expression of miR-361-3p and PTEN mRNA, whereas the high expression group showed the opposite trends (*P* < .05), as shown in Table 5.

**Table 5:** Comparison of lncRNA MIAT, miR-361-3p, PTEN, and cyclin D1 mRNA expression in different cell groups.

Group	lncRNA MIAT	miR-361-3p	PTEN	Cyclin D1
Negative control	1.00 ± 0.19	1.00 ± 0.17	1.00 ± 0.14	1.00 ± 0.10
miR-NC group	1.07 ± 0.16	0.99 ± 0.13	0.96 ± 0.19	1.05 ± 0.19
Low expression	0.61 ± 0.11*^,^†	1.43 ± 0.18*^,^†	1.82 ± 0.16*^,^†	0.59 ± 0.05*^,^†
High expression	1.73 ± 0.20*^,^†^,^‡	0.71 ± 0.09*^,^†^,^‡	0.73 ± 0.07*^,^†^,^‡	1.73 ± 0.10*^,^†^,^‡

lncRNA = long noncoding RNA, MIAT = myocardial infarction-associated transcript, miR = microRNA, PTEN = phosphatase and tensin homolog.

* Compared with the negative control, *P* < .05.

† Compared with the miR-NC group, *P* < .05.

‡ Compared with the low expression group, *P* < .05.

### 3.6. Assessment of cell proliferation, invasion, and apoptosis among different groups

**Figure 2.**
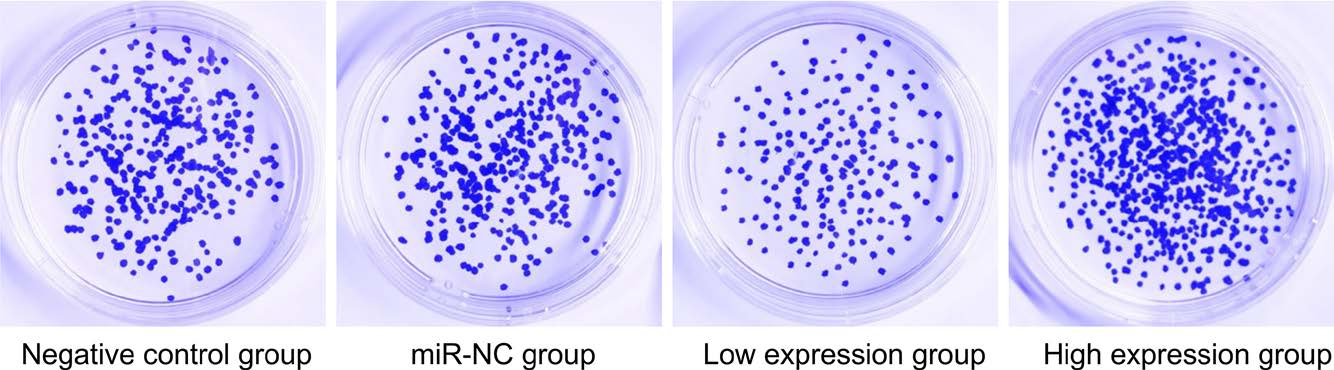
Comparative analysis of cell proliferation across different groups. miR = microRNA.

**Figure 3.**
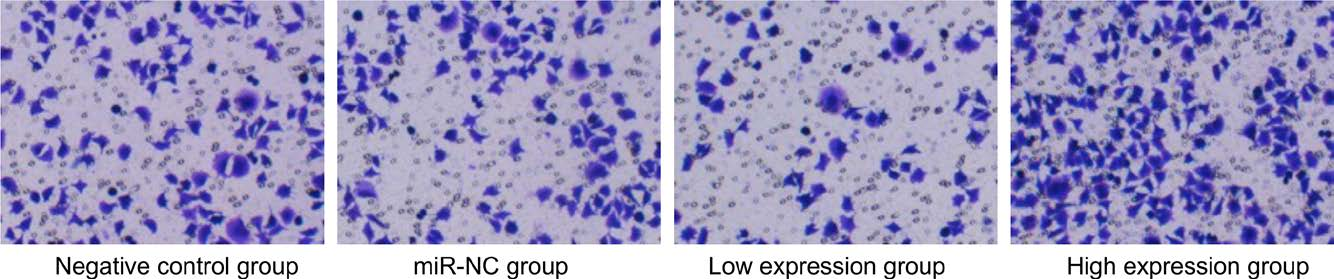
Comparative analysis of cell invasion across different groups. miR = microRNA.

**Figure 4.**
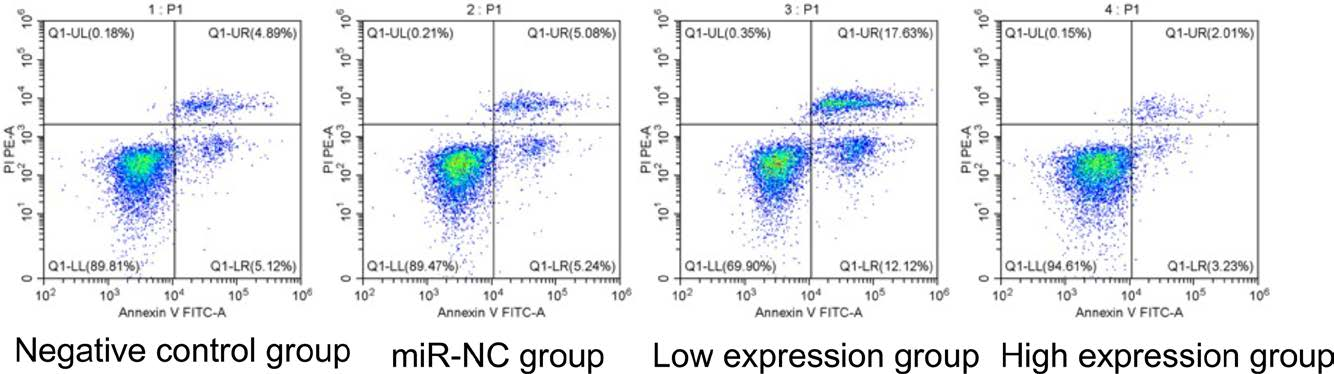
Comparative analysis of cell apoptosis rates across different groups. miR = microRNA.

No significant differences were observed in the negative control and miR-NC groups (*P* > .05). Compared with the negative control group, the low expression group exhibited a decrease in cell proliferation rate and invasion number and an increase in apoptosis rate. Conversely, the high expression group showed increased cell proliferation and invasion and decreased apoptosis (*P* < .05), as presented in Table 6 and Figures 2 to 4.

**Table 6:** Comparison of cell proliferation, invasion, and apoptosis rates among different groups.

Group	Proliferation rate (%)	Invasion number	Apoptosis rate (%)
Negative control	100.00 ± 16.71	573.59 ± 105.74	10.13 ± 2.51
miR-NC group	96.42 ± 7.28	562.41 ± 94.13	10.44 ± 1.82
Low expression	46.59 ± 9.23*^,^†	138.59 ± 43.85*^,^†	28.96 ± 4.73*^,^†
High expression	163.47 ± 20.44*^,^†^,^‡	1174.36 ± 275.41*^,^†^,^‡	3.76 ± 0.59*^,^†^,^‡

miR = microRNA.

* Compared with the negative control, *P* < .05.

† Compared with the miR-NC group, *P* < .05.

‡ Compared with the low expression group, *P* < .05.

## 4. Discussion

HCC has emerged as the second most common malignant tumor after lung cancer, primarily due to its invasive nature, high metastasis, and recurrence rates, representing a considerable hazard to public well-being. Patients with HCC typically exhibit a reduced rate of survival, often diagnosed at advanced stages with a lack of effective therapeutic targets. Studies on lncRNAs in various cancers indicate a strong association with HCC. LncRNA MIAT, for instance, is implicated in the pathogenesis of several cancers, including lung cancer, where it is upregulated.^[11]^ LncRNA MIAT is also involved in the progression of gastric, esophageal squamous cell carcinoma, HCC, and breast cancer^[12]^ and is reported to function as an oncogene promoting cell proliferation and invasion in HCC.^[13]^ However, the role of lncRNA MIAT in HCC has yet to be completely understood. This study confirmed significant upregulation of lncRNA MIAT in HCC tissues correlated with lymph node metastasis and advanced TNM stages. Silencing lncRNA MIAT in MHCC97L cells inhibited cell proliferation and invasion and induced apoptosis. The study also suggests that the effects of lncRNA MIAT on HCC cells might be mediated by regulating miR-361-3p, with the involvement of PTEN in this process.

The competing endogenous RNA hypothesis posits that lncRNAs can act as regulatory factors for active miRNAs. In search of downstream targets of lncRNA MIAT, our study identified potential binding sites through www.Targetscan.org and confirmed the interaction between lncRNA MIAT and miR-361-3p. Interestingly, the downregulation of lncRNA MIAT in prostate cancer cells (Du145) inhibited cell viability and induced apoptosis by upregulating miR-361-3p.^[14]^ Malignant tumors are characterized by sustained proliferation and evasion of apoptosis. Aberrant miRNA expression plays a role in regulating various cellular processes, often dysregulated in HCC. For example, upregulation of miR-221 in HCC increases cell proliferation and invasion, S phase cell count, and inhibits apoptosis.^[15]^ Downregulation of miR-361-3p in breast cancer and its restoration through inhibition of p21 protein-activated kinase 4 suppresses cell proliferation.^[16]^ Previous research and bioinformatics analysis indicate that miR-361-3p plays a significant and complex role in HCC development, suggesting dysregulation in HCC.^[17]^ Our results also demonstrate a reduction in miR-361-3p expression in HCC tissues and MHCC97L cells, aligning with previous studies.

PTEN, a downstream regulatory target of miR-361-3p, is pivotal in the development of tumors. Abnormal PTEN protein expression activates the PTEN/PI3K/Akt pathway, influencing the development of autoimmune diseases, diabetes, and other conditions.^[17]^ PTEN, a vital tumor suppressor gene, shows aberrant expression in cancers like gastric and colorectal cancer.^[18]^ Reduced or absent PTEN expression limits PIP3 dephosphorylation, preventing its conversion to PIP2. Accumulated PIP3 stimulates Akt activation in the PTEN/PI3K/Akt signaling pathway, triggering the expression of downstream molecules.^[19]^ Cyclin D1, an essential protein regulating cell proliferation during the G1 phase, and P27, a negative cell cycle regulator, interact to prevent cells from transitioning from G1 to the S phase, thereby inhibiting cell proliferation.^[20]^ Research has indicated that low miR-361-3p expression in liver cancer leads to decreased P27 and increased cyclin D1 expression, facilitating the transition of the cell cycle from the G1 to the S phase and enhancing liver cancer cell proliferation.^[21]^ Thus, overexpression of miR-361-3p can inhibit MHCC97L cell proliferation, induce G1/S phase arrest, and trigger apoptosis. Our results show that silencing lncRNA MIAT increases miR-361-3p and PTEN expression in MHCC97L cells, reducing cyclin D1 expression, inhibiting cell proliferation and invasion, and inducing apoptosis, while overexpression of lncRNA MIAT has the opposite effect.

In conclusion, lncRNA MIAT is significantly upregulated in HCC tissues, associated with lymph node metastasis and TNM staging. Silencing lncRNA MIAT can upregulate miR-361-3p expression, inhibit MHCC97L cell proliferation and invasion, and promote apoptosis, potentially providing new therapeutic targets for HCC treatment. However, utilizing lncRNA MIAT downregulation as a treatment strategy for HCC requires further extensive research for validation and support.

## Acknowledgments

The authors thank the staff of the Department of Gastroenterology at Shanghai Pudong New Area People’s Hospital and Shanghai Hongkou District Jiangwan Hospital for their technical support. This declaration is concise, comprehensive, and adheres to standard academic reporting guidelines.

## Author contributions

**Resources:** Hao Luo, Di Jin.

**Software:** Dongyun Hang, Di Jin.

**Validation:** Qingyu Wang.

**Visualization:** Qingyu Wang.

**Project administration:** Ming Xu, Liya Xu.

**Supervision:** Ming Xu, Liya Xu.

**Writing – original draft:** Jie Tang.

**Writing – review & editing:** Ming Xu, Liya Xu.
